# When Yawning Occurs in Elephants

**DOI:** 10.3389/fvets.2017.00022

**Published:** 2017-02-28

**Authors:** Zoë T. Rossman, Benjamin L. Hart, Brian J. Greco, Debbie Young, Clare Padfield, Lisa Weidner, Jennifer Gates, Lynette A. Hart

**Affiliations:** ^1^Department of Evolution and Ecology, University of California Davis, Davis, CA, USA; ^2^Department of Anatomy, Physiology and Cell Biology, School of Veterinary Medicine, University of California Davis, Davis, CA, USA; ^3^Department of Animal Science, University of California Davis, Davis, CA, USA; ^4^African Elephant Research Unit, Knysna Elephant Park, Western Cape, South Africa; ^5^Department of Population Health and Reproduction, School of Veterinary Medicine, University of California Davis, Davis, CA, USA

**Keywords:** yawning, elephants, contagious yawning, *Loxodonta africana*, *Elephas maximus*, elephant sleeping

## Abstract

Yawning is a widely recognized behavior in mammalian species. One would expect that elephants yawn, although to our knowledge, no one has reported observations of yawning in any species of elephant. After confirming a behavioral pattern matching the criteria of yawning in two Asian elephants (*Elephas maximus*) in a zoological setting, this study was pursued with nine captive African elephants (*Loxodonta africana*) at a private reserve in the Western Cape, South Africa, the Knysna Elephant Park. Observations were made in June–September and in December. In the daytime, handlers managed seven of the elephants for guided interactions with visitors. At night, all elephants were maintained in a large enclosure with six having limited outdoor access. With infrared illumination, the elephants were continuously recorded by video cameras. During the nights, the elephants typically had 1–3 recumbent sleeping/resting bouts, each lasting 1–2 h. Yawning was a regular occurrence upon arousal from a recumbency, especially in the final recumbency of the night. Yawning was significantly more frequent in some elephants. Yawning was rare during the daytime and during periods of standing around in the enclosure at night. In six occurrences of likely contagious yawning, one elephant yawned upon seeing another elephant yawning upon arousal from a final recumbency; we recorded the sex and age category of the participants. The generality of yawning in both African and Asian elephants in other environments was documented in video recordings from 39 zoological facilities. In summary, the study provides evidence that yawning does occur in both African and Asian elephants, and in African elephants, yawning was particularly associated with arousal from nighttime recumbencies.

## Introduction

Yawning is a widely recognized behavior in many mammalian and avian species and even some fish (1). In species with which we are familiar, yawning is basically self-evident; no one argues about whether a dog, cat, horse, rat, raccoon, or human is yawning when shown a photo or video clip, or seeing the behavior firsthand. But what about a species where the anatomy is considerably different from familiar mammals, and where a yawn may not be very evident even to experienced field investigators? We hypothesized that elephants may fall into this category. Prior to the initiation of our study, we were aware of no published report of yawning for any of the species of elephants in either a wild or captive/zoological setting.

Research on the function of yawning has increased markedly in the last decade or so, with several schools of thought about its function. Experimental evidence clearly shows that yawning does not influence blood oxygen or carbon dioxide levels (2–4). While there is some debate about the role of yawning in arousal (5), investigators generally agree about the association of yawning with brain activation (2–4, 6). Another proposed function of yawning, consistent with the brain activation role, is in cooling of the brain after its heating due to inactivity (7, 8).

If yawning does occur in elephants, one would expect a fixed-action pattern, as described in other species, with a slow opening of the mouth, followed by a brief frozen open posture, and then followed by a rapid, snap-like closure (1, 2, 6), with the size of the mouth opening varying from small to wide.

In this study, we first confirmed that elephants could perform an oral gaping behavior that met these descriptive criteria for yawning in pilot observations of African and Asian elephants at the San Diego Zoo and San Diego Zoo Safari Park. Once a behavioral pattern for elephant yawning was confirmed, we pursued a quantitative study of yawning in captive African elephants at a South African elephant park. Of particular concern was the occurrence of yawning as related to brain activation, particularly in the transition from sleeping or resting to wakefulness. Therefore, we focused on the likelihood of yawning in early morning hours compared with other times during the night. Because the study group was very stable, and we could observe the same individuals night after night, we looked for individual differences in the likelihood of yawning.

We also had an interest in whether or not a specific type of infrasonic vocalization occurred during yawning as in other circumstances, such as elephants greeting other elephants at waterholes or when leaving waterholes (9).

A particularly interesting aspect of yawning that has attracted considerable interest is so-called contagious yawning that is documented for humans, some non-human primates, wolves and budgerigars, and which is especially noteworthy among individuals that are familiar with each other (10–17). Contagious yawning is sometimes considered a manifestation of empathy, and elephants in nature form stable social groups, and their social empathic behavior is well documented (18). Therefore, we took the opportunity to observe the elephants for possible occurrences of contagious yawning.

Finally, the generality of yawning in both African and Asian elephants in other environments was investigated by examining video recordings from 39 zoological facilities in the U.S.

## Materials and Methods

### Approvals

For the pilot study, and the video recordings from the participating zoos, management at each zoo authorized the use of videos for this study. The Zoological Society of San Diego’s Institutional Animal Care and Use Committee approved the protocol on behalf of all participants (N.I.H. Assurance A3675-01; Protocol 11-203). The UC Davis Institutional Review Board declined review of the protocol because it was only observational with no intervention. Permission to conduct the observations of elephants at Knysna Elephant Park in South Africa was granted to the investigators by park management, and the on-site African Elephant Research Unit oversaw the research we conducted there.

### Pilot Observations of Asian Elephants at the San Diego Zoo and San Diego Zoo Safari Park

Elephants were observed at both facilities in the outdoor exhibit spaces between dawn and dusk. Instances of oral gaping behaviors that met the criteria of slow opening of the mouth, followed by a brief frozen open posture, followed by a rapid, snap-like closure were noted, and if possible, video recorded. Observations were made on 6 Asian elephants (1 male and 5 females) at the San Diego Zoo and on 13 African elephants (5 males and 8 females) at the San Diego Zoo Safari Park. Brian J. Greco photographed or video recorded oral gaping behaviors meeting the criteria for yawning in five Asian elephants (one male and four females) and two African elephants (one male calf and one adult female).

### Observations at the South African Location

After the general pattern of yawning in elephants was verified through the review of the videos of yawning at the San Diego Zoo, a study was launched at Knysna Elephant Park, Western Cape in South Africa, a private reserve maintained as an attraction for tourists to experience supervised contact with elephants. Most observations were during June–September, the cooler season, and additional observations were made in the warmer season during December, 2015. The subjects were nine African elephants which were followed individually with regard to yawning: four adult females, Sally 25 years, Nandi 22 years, Thandi 12 years, Keisha 11 years; one sub-adult female Thato 7 years; two sub-adult males, Shungu 8 years and Mashudu 7 years; and two adult males, Clyde 22 years and Shaka, 14 years.

In daytime observations over 34 days, seven of the elephants, excluding the adult males, were observed as they moved about and when being watched by visitors. The adult males were kept in a separate location during the daytime. Daytime hours were defined as the period where the elephants were in the field available for tourist interaction, generally from 8:45 a.m. to 4:45 p.m. During the visitation periods, one investigator (Zoë T. Rossman) observed the elephants for all occurrences of yawning during 2-h sampling periods, in the morning at various times, and again in the afternoon, at various times. Daytime observations covered a total of 273 h. The occurrences of yawning in the daytime are not presented on a per-elephant basis because of the rarity of yawning.

In 47 nighttime observations from 6:00 p.m. to 6:30 a.m., all nine elephants were kept within a large darkened open-air enclosure (14 m × 14 m), illuminated with infrared lighting and continuously recorded by two stationary Hikvision low-light video cameras and a DVR system with motion detection. The videos were reviewed with the Hikivision iVMS-4200 software (DVR model DS-7116HWI-SL, camera model DS-2CE1582P-VFIR3). Six elephants could walk freely between the enclosure and a fenced area outside (2 ha). The other three elephants (two adult males and one adult female) were enclosed in stalls (6 m × 4 m) in the back of the enclosure and were also visible on the two video cameras. In June–September, the enclosure was warmed by heat lamps, so the ambient temperature in the enclosure was warmer than outdoors, and comparisons between the two seasons with regard to likelihood of yawning therefore were not feasible. That said, in June–September, the mean nighttime temperature in the enclosure was 10°C, and in December, the mean nighttime temperature was 17°C.

While the elephants could walk outside in the fenced area, they spent most of the night in the enclosure and slept or rested in a recumbent manner at least once each night, with 1–3 recumbent bouts per elephant per night being typical. A recumbent bout was defined as the entire period when an elephant was laying down until the elephant arose from the recumbency and was standing. The bout durations were recorded. However, not all arousals from a recumbency were spontaneous, and sometimes an elephant could be aroused from a recumbent bout by another elephant arousing from a recumbency or another elephant walking by. Final bouts, defined as the last bout of the night for each elephant, generally occurred 1–3 h before elephants were led outdoors by guides for daytime activities. Non-final bouts are all other recumbent bouts for each night.

Through preliminary observations of the night video recordings during the first week of the study, it became apparent that almost all yawning occurred in association with an arousal from a recumbent bout. Thus, particular attention was paid to the periods immediately before and during an arousal from the recumbent bout and during the 2 min after standing.

In viewing the nighttime video recordings, the videos were viewed in fast forward except when an arousal from a recumbency was noticed, in which case the times immediately before, during, and 2 min after an arousal were viewed for the occurrences of yawning. When a yawn occurred, the duration was recorded. Clipped sections of videos where yawns occurred were downloaded and saved for later review as needed. The individual performing each yawn was recorded, so that we could examine for differences in likelihood of yawning. Periods where an elephant was facing away from the cameras were not assessed as to whether a yawn occurred. There were control nights where the videos for the entire night were scanned in fast forward for the occurrence of yawns aside from those associated with recumbencies. Two investigators (Zoë T. Rossman and Lisa Weidner) examined the video records for occurrences of yawning.

Given the use of heat lamps for warming the nighttime enclosure during the months of June–September, and the lack of feasibility of accurately comparing data between seasons, the data for the two seasons are combined for presentation. There were 545 h of video recordings that were examined over 47 nights for the two seasons for the occurrences of yawns during arousals from recumbencies. In addition, there were 72 h of nighttime video recordings for control observations during periods not associated with recumbencies that were reviewed for the occurrence of yawns.

The methods described here were designed to address a prediction of the hypothetical function of yawning, that of activation or arousal. In the case of this study, the prediction would be that yawning would be more likely in association with arousals from the last recumbencies in the morning before leaving the enclosure than with arousals from non-final recumbencies.

Vocalizations were recorded in conjunction with nighttime video recordings to determine whether the elephants were producing some infrasonic vocalizations in association with yawning. Acoustic recordings with live monitoring were made in December over five nights from 11 p.m. through 6 a.m. in the enclosure at a distance from the elephants ranging from 10 to 30 m. These acoustic recordings could then be reviewed in a time-synchronized manner with the video recordings to determine if vocalizations (infrasonic) did occur at night and if any vocalizations were temporally associated with yawning. The recording equipment was a Sanken CS-1 directional condenser microphone [flat frequency response = 50–20,000 Hz; sensitivity = −30dB/Pa ± 1dB (31.6 mV)] and a Fostex FR-2 digital field recorder (resolution = 16 bits; sampling rate = 44.1 kHz). Time-labeled recordings were imported into MacBook laptop and iMac desktop computers in wav files for review.

Finally, at the South African location, occurrences of possible contagious yawning were also of interest in viewing the video recordings. Aside from illustrations showing overlapping yawns by two individuals, we are unaware of any published criteria for designating a contagious yawn, based on the interval between the start of a yawn by one individual and the start of a yawn by another individual and/or the degree of overlap in the yawns. In this study, only when the two elephants were in a position where yawns could be discerned on the video recordings could a determination be made as to whether or not a contagious yawn had occurred. Yawns that were overlapping, or closely spaced together, were considered contagious, and the elephant initiating a yawn second was considered the one yawning contagiously. In order for a yawn to be considered contagious, the elephant yawning contagiously needed to be in a position to have had direct visibility of the yawn of the first elephant. With the video recording played back in time-lapse, the delay from the start of a yawn by one elephant to the start of a yawn by the other elephant and the time of overlap were noted when the times were discernable. For yawns that were not overlapping, we used an interval of 10 s after the finish of a yawn by one elephant to the start of a yawn by the other elephant as the cutoff for labeling the yawn of the observing elephant as contagious. We also identified the individual elephants involved in the contagious yawning so as to specify the sex and age categories of the pairs involved.

### Study of Yawning in African and Asian Elephants at 39 Zoo and Aquarium Facilities

One investigator (Jennifer Gates) reviewed videotapes for oral gaping behaviors in 300 h of video recording from 30 elephants (African: *N* = 8 males, 5 females; Asians: *N* = 12 males, 5 females) housed at Association of Zoos and Aquariums-accredited facilities. Particular attention was paid to recordings of oral gaping behaviors meeting the characteristic pattern of yawning: a slow opening of the mouth, followed by a brief frozen open posture, followed by a rapid, snap-like closure. The video recordings were collected for the behavior portion of the Elephant Welfare Project, involving elephants housed at 39 Association of Zoos and Aquariums facilities (19).

### Statistical Analyses

We tested the prediction that yawns would most likely occur toward the morning hours in association with the last recumbent bout of the night in comparison with non-final recumbencies. For this comparison, a chi-square test with 1° of freedom was used. To test for individual differences in yawning frequency, the Genmod procedure for least squared means was used.

## Results

### Pilot Observations at the San Diego Zoo and San Diego Zoo Safari Park

**Figure 1:**
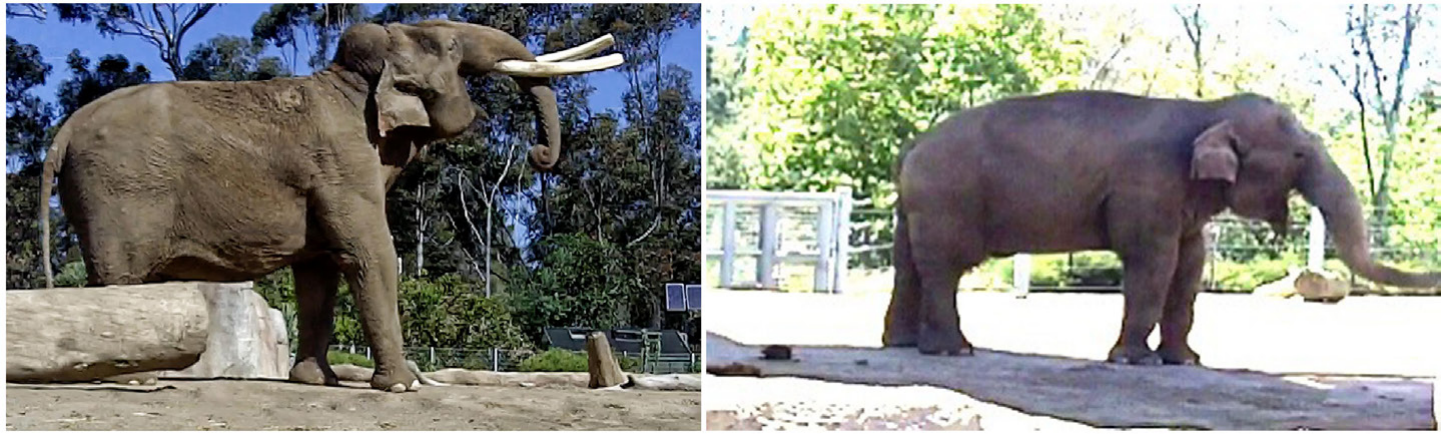
**Yawning in two Asian elephants taken at the San Diego Zoo Safari Park**. The video clips with these images are available in Supplementary Material (with permission of San Diego Zoo Global).

After viewing photo and video examples of yawns, the care staff at both facilities reported observing other elephants performing yawns; these observations were not recorded. Figure 1 and Video S1 in Supplementary Material show clear examples of yawns in one male and one of the female Asian elephants during midday daylight hours. The yawn of one elephant was uncharacteristically pronounced, as compared with subsequent observations conducted in South Africa.

### Daytime Observations in South Africa

Yawning was a rare occurrence in the daylight hours. In the June–September season with 243 h of daytime observations on the seven elephants, only one yawn was observed. This was seen after the elephant aroused from a recumbent bout. In December, with 30 h of daytime observation on the same elephants, no yawns were seen.

### Nighttime Video Observations in South Africa

#### Recumbent Bouts

In the enclosure in the evening, the elephants typically fed and moved about until approximately midnight. Then, for the rest of the night they would lie down and rest or sleep in sporadic bouts of 1–3 h in what are referred to as recumbent bouts or just recumbencies. During the 47 nights of observations over both seasons, each of the elephants had at least one recumbent bout, with a mean of 92% of the nights and an overall mean of 2.5 bouts per elephant per night.

From viewing the video records for all elephants, a total of 1,051 visible arousals from recumbencies were seen with a mean recumbent bout duration of 78 min.

The total number of recorded final recumbent bouts per elephant ranged from 14 to 24, and the total number of non-final bouts recorded per elephant ranged from 8 to 48 (Table 1). The duration of final bouts for each elephant ranged from a mean of 76 to 127 min, and the duration of non-final recumbent bouts ranged from a mean of 51 to 104 min (Table 1). A recumbent bout sometimes appeared to be terminated when an adjacent elephant was arousing from a recumbency or when another elephant was walking around. Therefore, it was not feasible to test for differences in bout durations as a function of being a final or non-final bout.

**Table 1:** **The occurrences of recumbent sleeping/resting bouts and bout durations in the nighttime observations in the Knysna Elephant Park in southern South Africa**.

Elephant name	Total # final recumbent bouts	Total # non-final recumbent bouts	Mean duration final recumbent bouts (min)	Mean duration non-final recumbent bouts (min)
Sally	19	48	76	72
Nandi	24	38	78	63
Thandi	14	31	107	81
Keisha	20	39	107	64
Shungu	17	32	98	51
Mashudu	16	19	109	63
Thato	20	24	127	80
Shaka	16	15	121	104
Clyde	14	8	93	63
Mean	18	28	102	71

*The data include observations over a total of 47 nights*.

#### Yawning

As with the daytime observations, nighttime yawning was a rare occurrence in the enclosure when the elephants were standing or walking around, with just four yawns seen in 74 h of control nighttime observations. Because a yawn was only visible when the elephant was in a frontal or side view, some yawns while the elephants were standing could have gone unnoticed.

**Figure 2:**
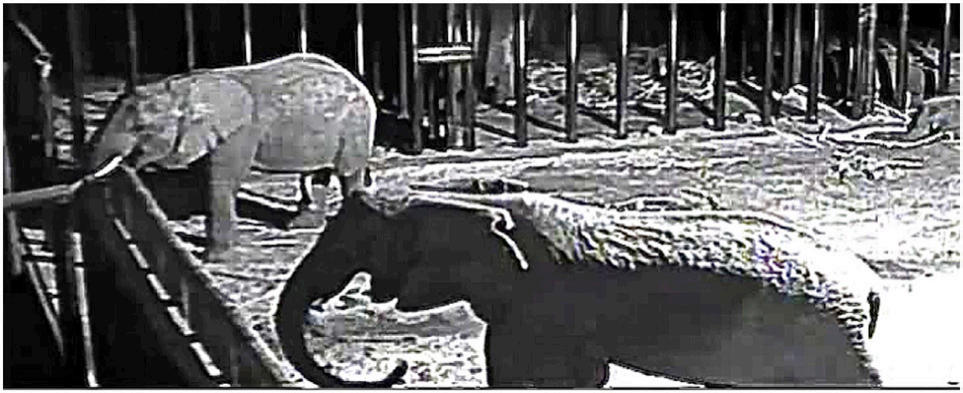
**Contagious yawning in African elephants at the Knysna Elephant Park in southern South Africa**. The adult female, Keisha in the foreground, who had just stood up, starts yawning 2 s after the sub-adult male, Mashudu, in the background, begins yawning. Mashudu had just previously stood up from a recumbency. See Table 3, Set 1 for details. The video clip with this image is available in Supplementary Material (with permission of the Knysna Elephant Park).

Almost all yawns were observed in association with arousals from a recumbency. Across all arousals from recumbencies, 133 yawns were recorded. Examples of such yawns are shown in Figure 2 and in sample video clips of yawns in Supplementary Material.

As shown in Table 2, the occurrences of final recumbencies per elephant where a yawn was observed ranged from 26 to 94% with a mean of 54% across all elephants. The percentages of non-final bouts where a yawn occurred ranged from 3 to 63% per elephant, with a mean of 19% across all elephants. The percentage of final bouts with a yawn was significantly higher than the percentage of non-final bouts with a yawn (*p* < 0.0001, chi-squared statistic, 24.97). The mean duration of yawns associated with the final bout was 6 s, which was the same as the mean duration of yawns associated with the non-final bouts (Table 2). During the 47 nights of observations, all elephants were seen to yawn on at least some nights; however, not every elephant was observed yawning every night. When elephants did yawn, the number of yawns ranged from 1 to 4 yawns per night.

**Table 2:** **The occurrences of yawns and yawn durations associated with arousals from the final recumbent bouts and the non-final bouts in the nighttime observations in the Knysna Elephant Park in southern South Africa**.

Elephant name	Total # yawns in final bout (%)	Total # yawns in non-final bout (%)	Mean duration of yawns in final bouts (s)	Mean duration of yawns in non-final bouts (s)
Sally	5/19 (26%)	6/48 (13%)	5	6
Nandi	16/24 (67%)	4/38 (11%)	7	7
Thandi	5/14 (36%)	4/31 (13%)	6	4
Keisha	12/20 (60%)	11/39 (28%)	8	7
Shungu	5/17 (29%)	1/32 (3%)	6	5
Mashudu	15/16 (94%)	8/19 (42%)	5	9
Thato	13/20 (65%)	4/24 (17%)	6	4
Shaka	5/16 (31%)	4/15 (27%)	5	NA^a^
Clyde	10/14 (71%)	5/8 (63%)	6	9
Mean	53%	24%	6	6

*The data include observations over a total of 47 nights. The first column shows for each elephant the number of yawns in the final bouts divided by the total number of final bouts and the percentage of bouts with a yawn. The second column shows the total number of yawns in non-final bouts divided by the total number of all non-final bouts and percentages with a yawn. There were significant individual differences in overall yawning frequency with Mashudu yawning the most and Sally yawning the least (see text for details)*.

*^a^Yawn durations were not visible*.

There were significant differences among elephants in the likelihood of yawning. In the least squared means test, Mashudu was significantly more likely to yawn overall than others (*p* = 0.035, *z* 2.11) and those less likely to yawn than others were Sally (*p* ≤ 0.0001, *z* −4.38), Shungu (*p* = 0.0002, *z* −3.76), Thandi (*p* = 0.0012, *z* −3.23), Shaka (*p* = 0.024, *z* −2.26), and Nandi (*p* = 0.034, *z* −2.12). The mean individual yawning frequencies are shown in Table 2.

#### Vocalizations during Yawning

Acoustic recordings showed that the elephants vocalized at times during each night. Thus, we confirmed that the acoustic recording system detected vocalizations throughout the recording distances. A review of time-synchronized acoustic and video recordings, however, revealed that no vocalizations were associated with yawns.

#### Contagious Yawning

We observed six instances of what we considered to be contagious yawning. There was one instance of yawning by a standing elephant after seeing an arousing elephant yawning and there were five instances of yawning by elephants arousing from recumbencies in association with seeing another arousing elephant yawning. The details regarding the sexes and ages of the pairs of elephants engaged in contagious yawning are given in Table 3. The contagious yawning seen in pairs of elephants arousing from recumbencies were associated with the last recumbencies of the night. The observations were too few to determine if some elephants engaged in contagious yawning more than others. We did, however, document that contagious yawning occurred both in pairs of like and unlike sexes and in pairs of like and unlike age categories (adults or sub-adults). An image of the contagious yawn by a standing elephant is shown in Figure 2 and in a video clip S2 in Supplementary Material. When detailed timing could be discerned, the delay between the start of a yawn by one elephant and the start of a yawn by the other elephant is given as well as the time of overlap, if the yawns did overlap.

**Table 3:** **Details of the six postulated contagious yawning episodes meeting specified criteria (see text) in nighttime observations at the Knysna Elephant Park in southern South Africa**.

*Contagious yawning in a standing elephant. N = 1*
One sub-adult male (Mashudu) and one adult female (Nandi). Nandi had been standing for over 5 min. Mashudu, initially lying down, starts standing and then yawns while starting to stand. Nandi then starts yawning 18 s later with no overlap in yawning times. Occurred in association with the final recumbency for Mashudu
*Contagious yawning in arousing elephants, N = 5*
Set 1. One sub-adult male (Mashudu) and one adult female (Keisha). Both elephants initially lying down. Mashudu stands first, and then Keisha starts standing. Mashudu starts yawning, and Keisha starts yawning 2 s later with a 2 s overlap in yawning times. Occurred on final recumbency for both elephants (Illustrated in Figure 2 after both are standing)
Set 2. Two adult females (Nandi and Thandi). Both elephants initially lying down, then both start standing. Nandi starts yawning, and Thandi then starts yawning 5 s later, with an 8 s overlap. Occurred on the final recumbency for both elephants
Set 3. One adult female (Nandi) and one sub-adult male (Shungu). Both elephants initially lying down, then both start standing. Nandi starts yawning, and Shungu then starts yawning 6 s later with a 3 s overlap. Occurred on the final recumbency for both elephants
Set 4. Two females (sub-adult female Thato and adult female Keisha). Both elephants initially lying down, then both start standing. Keisha starts yawning, and then Thato starts yawning 16 s later, with no overlap in yawning times. Occurred on the final recumbency for both elephants
Set 5. Two males (sub-adult Mashudu and adult Clyde). Both elephants initially lying down, then both start standing. Mashudu yawns, and then Clyde yawns. Information on delay between the starting of yawning and overlap times could not be discerned. Occurred on the final recumbency for both elephants

### Study of Yawning in African and Asian Elephants at 39 Zoo and Aquariums Facilities

We observed 19 elephants (African: *N* = 1 male, 2 females; Asian: *N* = 8 males, 8 females) performing oral gaping behaviors meeting the criteria for yawning. Of the 38 yawns we observed, 14 occurred between 1 a.m. and 7 a.m., and 26 occurred between 12 p.m. and 7 p.m. (the time period when most recordings were made). The elephants also performed oral gaping behaviors when feeding and when performing stereotypic behaviors. But, these gaping behaviors differed from the characteristic pattern of yawning. For example, oral gaping in the context of feeding occurred prior to food being placed into the mouth or when chewing, and the opening and closing of the mouth did not fit the pattern of yawning and typically involved some lateral jaw movement. When performing stereotypic behaviors, some elephants displayed a slack jawed posture, where the mouth was held slightly open, as if it were relaxed.

## Discussion

To our knowledge, this is the first data-based report of the occurrence of yawning in African and Asian elephants. The observations reveal that yawning in African elephants at the South African study site was a pervasive behavior that occurs in a very specific context—that is, in association with arousals from nighttime recumbent sleeping/resting bouts. Using observations from this well-established group of nine elephants in a South Africa reserve, where at night they were maintained in an open-air enclosure with infrared video recording, we were able to compile data on virtually all yawning episodes during the study periods. This unique opportunity allowed us to document which elephants yawned the least and the most, and when yawning was most likely to occur. There was no age or sex pattern with regard to which elephants yawned the least or the most. For example, of the two sub-adult males, Mashudu yawned the most, and Shungu was one of those yawning the least.

Of the 133 yawns associated with arousals from recumbencies, over half occurred in association with an arousal from the final recumbency of the night, just prior to the morning. There were significantly fewer yawns, per elephant, associated with arousals from 1 to 3 nightly non-final recumbencies. This finding is consistent with our prediction from the brain activation theory of yawning, proposed by others (2–4, 6). The recording of infrasonic vocalizations in the enclosure at night revealed that while the elephants did vocalize from time to time, there were no evident vocalizations associated with yawning.

The degree to which the pattern of yawning we describe here might apply to wild free-ranging African elephants remains to be seen. Such observations would be difficult because they would be expected to occur primarily between midnight and early morning when visibility would be restricted and the observation distance would likely be much greater. No vocalizations would be expected to indicate that an elephant was yawning. That said, the generality of yawning in both African and Asian elephants in both sexes was evident in examination of video recordings from North American Zoos where 38 yawns were observed in male and female African and Asian elephants.

With the opportunity to observe a well-integrated herd of elephants throughout the night, *via* video recordings, we observed five instances of what appeared to be contagious yawning in pairs of elephants arousing together from recumbent bouts, in which the yawning was usually overlapping but differed in start times, and one instance when a standing elephant yawned after observing another arousing elephant that yawned. The contagious yawning episodes seen in pairs of elephants arousing together all occurred in the final recumbencies of the night, suggesting an association with the brain activation theory of yawning that may even be socially facilitated. We were able to establish that contagious yawning occurred in pairs of the same or different sexes and in pairs of the same and different age categories (adults and sub-adults). While our designation of some yawning episodes as contagious is consistent with descriptions of contagious yawning in other species (10–17), the findings are unique with regard to individual details pertaining to the contagious yawning pairs. As with the occurrence of yawning in general, observations on yawning in an integrated group of elephants in natural settings are needed to reliably establish the occurrence and context of contagious yawning in this species.

## Conclusion

In addition to being useful in understanding the occurrence of yawning as an essential aspect of elephant behavior, this study adds an important comparative component for current research on the brain activation function of yawning. Elephants now represent the first megafauna species with data on when yawning occurs and some variables that may influence yawning. In addition, our finding of likely occurrences of contagious yawning in elephants adds to a comparative perspective on this aspect of yawning.

## Author Contributions

Conceived and designed study; drafted and compiled manuscript: BH, LH, ZR, BG, DY, and CP. Collected and compiled and analyzed data: ZR, LW, BG, JG, BH, and LH.

## Conflict of Interest Statement

The research was conducted in the absence of any commercial or financial relationships that could be construed as a potential conflict of interest.

## Supplementary Material

Video S1 **A yawn by an Asian elephant, atypically wide, at the San Diego Zoo Safari Park**.

Video S2 **Taken at night at the Knysna Elephant Park in South Africa shows an elephant arousing and yawning, contagiously, after seeing the elephant in the background starting to yawn**.
